# Identification of *Rcr12*, a single dominant clubroot resistance gene near *Rcr6* on chromosome B3 of *Brassica nigra*

**DOI:** 10.1186/s12870-025-06947-3

**Published:** 2025-07-18

**Authors:** Hao Hu, Adrian Chang, Ling Cao, Yangdou Wei, Fengqun Yu

**Affiliations:** 1 Saskatoon Research and Development Centre, Agriculture and Agri-Food Canada, 107 Science Place, Saskatoon, Saskatchewan, S7N 0X2 Canada; 2 Department of Biology, University of Saskatchewan, Saskatoon, Saskatchewan, S7N 5E2 Canada

**Keywords:** Clubroot resistance, *Brassica nigra*, *Plasmodiophora brassicae*, Bulked Segregant RNA-sequencing, Single protoplast isolates, SNP markers, NLR gene cluster, Pangenomics

## Abstract

**Background:**

Clubroot disease, caused by the soil-borne protist *Plasmodiophora brassicae*, is a major threat to *Brassica* crops worldwide, leading to significant yield losses. Genetic resistance is the most effective and sustainable management strategy; however, the identification and characterization of clubroot resistance (CR) genes remain a challenge, particularly in *Brassica nigra*. Despite its abundant CR resources, only one CR gene, *Rcr6*, has been identified in the B genome of *B. nigra*, leaving much of its genetic potential unexplored. Understanding the genomic distribution and diversity of CR genes in *B. nigra* is crucial for expanding resistance breeding options, especially for canola (*B. napus*).

**Results:**

This study identified *Rcr12*, a single dominant CR gene on chromosome B3 of the highly resistant *B. nigra* line BRA19278. Using bulked segregant RNA sequencing (BSR-seq) and fine mapping in segregating populations derived from a cross between CR2748 (a susceptible *B. nigra* line) and BRA19278, together with *s*ingle-root *p*rotoplast-derived *i*solates (SPIs) of *P. brassicae* and comparative analysis across multiple reference genomes, we established that *Rcr12* is distinct from *Rcr6* despite their close physical proximity. Evidence supporting its distinctiveness includes differential resistance patterns against various SPIs, unique SNP marker associations, and pangenomic analyses. Fine mapping refined the *Rcr12* locus to a 0.33 Mb region on chromosome B3, containing multiple resistance gene candidates, in contrast to the single candidate identified for *Rcr6*. This study is the first to report an nucleotide-binding leucine-rich repeat (NLR) cluster-type CR locus near an NLR singleton in *Brassica* crops, underscoring the evolutionary and functional significance of this gene arrangement.

**Conclusion:**

The discovery of *Rcr12* expands our understanding of NLR gene organization and its role in host resistance evolution. Beyond advancing clubroot resistance breeding, this discovery lays the groundwork for studying functional interactions between NLR singletons and clusters in plant immunity. Additionally, the use of purified SPIs as a pathogen differentiation tool offers a novel approach to resolving ambiguities in clubroot research, addressing the complexity of host-pathogen interactions and facilitating future investigations, especially with the anticipated release of a new pathogen classification system.

**Supplementary Information:**

The online version contains supplementary material available at 10.1186/s12870-025-06947-3.

## Introduction

Clubroot, induced by the protist pathogen *Plasmodiophora brassicae* Woronin, represents a severe affliction primarily targeting *Brassica* crops. *P. brassicae* triggers the formation of enlarged, club-shaped roots, disrupting water and nutrient uptake in infected plants. Initially detected in Alberta, Canada in 2003, clubroot swiftly disseminated throughout the prairie provinces in Western Canada. It is estimated that the disease leads to a 10–15% reduction in yield for brassica crops [1], including the vital oilseed crop canola (*Brassica napus* L.) in Canada. Developing canola cultivars endowed with genetic resistance stands as the most effective and economical strategy for disease management [2, 3]. Over the past three decades, the identification of clubroot resistance (CR) genes has been a focus within crops related to canola in the *Brassicaceae* family. This family includes the Triangle of U, encompassing diploid species such as *B. rapa* (genome AA, *n* = 10), *B. nigra* (BB, *n* = 8), and *B. oleracea* (CC, *n* = 9), as well as amphidiploid species like *B. napus* (AACC), *B. juncea* (AABB), and *B. carinata* (BBCC). Notably, the majority of reported CR genes have been identified within the A genome. Specifically, *CRa* [4, 5], *CRb* [6], *CRb*^Kato^ [7, 8], *CRd* [9], *CRk* [10], *Crr3* [11], *Rcr1* [12, 13], *Rcr2* [14], *Rcr4* [15], *Rcr5* [16], *Rcr10*^ECD01^ [3], and several quantitative trait loci (QTLs) [17–20] have been identified from chromosome A03. Similarly, *Crr1* [21, 22], *CRs* [23], *Rcr3* [24], *Rcr9* [15], *Rcr9*^Wa^ [24], *Rcr9*^ECD01^ [3], *Rcr9*^ECD02^ [25], and several QTLs [17, 19, 20] have been located on chromosome A08. Conversely, a smaller number of CR genes/QTLs have been identified on chromosomes A01 [17, 18, 22], A02 [10, 15], A04 [18], A05 [18], and A06 [26]. Within the C genome, the majority of CR resources identified are QTLs [18, 27, 28], with one major CR gene, *Rcr7*, located on chromosome C7 [29]. However, in contrast to the abundance of CR resources found within the A and C genomes, only one major CR gene, *Rcr6*, has been reported in the B genome [30], indicating a lack of exploration in *B. nigra* materials for clubroot resistance.

The reported CR genes from *Brassica* crops exhibit two patterns. First, most mapped CR genes are clustered on aggregated regions of selected chromosomes. Second, most functionally-confirmed CR genes encode nucleotide-binding leucine-rich repeat (NLR) proteins. Regarding the clustering of CR genes, studies have reported a clear aggregation of mapped CR loci on chromosomes A03 and A08 [31, 32]. Integrated CR loci, such as *CRA3.7* and *CRA8.2*, have been proposed to represent CR genes clustered in closely adjacent regions [32]. For instance, *CRA3.7* integrates CR loci spanning the 24.95–25.14 Mb region of chromosome A03 in the ECD04 reference genome [32]. This region includes three reported CR loci, namely *Rcr1*, *Rcr2*, and *CRa*, identified from three *Brassica* vegetables (*B. rapa*). Through cloning efforts, the three reported CR loci (i.e., *Rcr1*, *Rcr2*, and *CRa*), and the functionally-verified *CRA3.7.1* (*BraA03g012133E*) of *CRA3.7* were found to share identical sequences in their coding regions [31, 32]. However, from an adjacent integrated locus *CRA3.4* (22.66–24.54 Mb on chromosome A03), *Rcr4* was found to have an identical coding sequence with the *Rcr1* group, while *Rcr5* was not [31]. Furthermore, in the *CRA3.7* locus, whether the other four NLR genes arranged in tandem repeated organization with *CRA3.7.1* (i.e., *CRA3.7.2* to *CRA3.7.5*) are functional CR genes have not yet been studied. These uncertainties over the clustered CR genes and the integrated CR loci all warrant further investigation. Gene clustering is uncommon in eukaryotes; however, NLR-type disease resistance (*R*) genes often cluster in plant genomes [33]. This clustering phenomenon is more prevalent in higher plants compared to early plant lineages, suggesting an evolutionary advantage [33, 34]. In contrast to NLR singletons (individual NLR genes whose function does not rely on additional NLRs), clustered NLRs can operate as more complex systems, often referred to as NLR networks, which combine a higher degree of evolvability with functional robustness. The functional and evolutionary connections between these two types of NLR organizations are still poorly understood and require further exploration [34].

Regarding the identity of CR genes, the four cloned and functionally verified CR genes, i.e., *Rcr1*/*CRa*/*Crr1a/BjuA03.BNT1*, all encode Toll/interleukin-1 receptor (TIR)-NB-LRR (TNL) proteins [4, 31, 32, 35]. TNL proteins, one of the two main subfamilies of NLRs, execute distinct plant immune responses compared to coiled-coil (CC)-NB-LRR (CNL) proteins [36]. These canonical TNL proteins typically function as intracellular immune receptors, recognizing pathogen effectors with race-specificity. Upon activation by pathogen effectors, TNL proteins can reorganize to form oligomers (i.e., holoenzyme complexes) and catalyze the generation of immune-related small molecules that trigger downstream immune responses [36–38]. Nonetheless, other types of *R* genes should not be disregarded as CR gene candidates. For instance, a novel CR gene, *Weitsing*, identified from *Arabidopsis* was found to encode a small protein (i.e., WTS) that could self-assemble to form an endoplasmic reticulum (ER)-localized pentamer Ca^2+^-permeable channel. Unlike NLR type R proteins with race-specificity, WTS requires no pathogen effectors in its polymerization process and thus confers broad-spectrum resistance to *P. brassicae* [39]. Besides, *R* genes encoding receptor-like proteins (RLPs) and receptor-like kinases (RLKs) that are commonly involved in pathogen-associated molecular pattern (PAMP)-triggered immunity (PTI) are yet to be confirmed in the CR gene family. Therefore, it could be anticipated that more varieties of CR genes will be revealed in the future.

In this study, we investigated *B. nigra* material BRA19278, which exhibited a strong yet distinct CR phenotype compared to the previously reported *B. nigra* material PI219576 carrying *Rcr6*. We confirmed that the CR phenotype in BRA19278 is controlled by a single dominant gene, *Rcr12*. Bulk segregant analysis (BSA) was performed using RNA-seq data from resistant (R) and susceptible (S) plant bulks of a BC_1_ population derived from the cross between BRA19278 and the susceptible CR2748 (*B. nigra*). Through fine mapping, we developed several new SNP markers, narrowing down the *Rcr12* target region to 0.33 Mb on chromosome B3. This refined region contains multiple candidate NLR-type *R* genes and overlaps slightly with the region harboring *Rcr6*. However, distinct resistance specificities against *P. brassicae* isolates and pan-genomic analysis using multiple reference genomes confirmed that *Rcr12* is a separate CR gene from *Rcr6*. Additionally, this study marks the first report of an NLR cluster-type CR locus near another singleton CR gene in *Brassica* crops, highlighting the evolutionary and functional significance of this unique gene organization.

## Materials and methods

### Plant materials and growth conditions

Seeds of *B. nigra* lines BRA19278 (CR, male parent) and CR2748 (clubroot susceptible, female parent) were obtained from Nutrien Ag Solutions (Saskatoon, Canada) and IPK (Leibniz Institute of Plant Genetics and Crop Plant Research, Seeland, Germany), respectively. The CR line BRA19278 was crossed with the susceptible female line CR2748 to produce the F_1_ progeny. The F_2_ population was generated via self-pollination of F_1_ plants, while the BC_1_ population was made by back-crossing resistant F_1_ plants with the female parent CR2748.

ACDC is a clubroot-susceptible canola line (*B. rapa*) and was included as a susceptible control. Seeds of the *Rcr6*-bearing *B. nigra* line PI219576 (CR) were also provided by Nutrien Ag Solutions [30]. Y549-(0)−2-1 is an introgression line between *Rcr1*-bearing pak choy cultivar Flower Nabana (*B. rapa* subsp. *chinensis*) and an elite canola line DH16516 (*B. napus*), which has reached homozygous status at the *Rcr1* locus after generations of backcrossing/selfing and selection (at least BC4S2 generation) [31]. HH393-2 is a transgenic canola line carrying *Rcr1* gene (homozygous status) developed with our fast-breeding system [31], and DH12075 (*B. napus* L.) was the elite canola line used for this transformation. These plants were included as controls to demonstrate the ability of different *P. brassicae* pathotypes/isolates to differentiate resistance responses. All plants were grown in a growth chamber under long-day conditions (i.e., 16/8 hours of light/dark, light strength of 230 µmol m^–2^ s^–1^ at the canopy level) at a constant 22℃.

### Clubroot pathogenicity tests

Clubroot pathogenicity tests were performed as described [15], and two types of inoculums of *P. brassicae* were used in this study. One type of inoculum was the field-collected strains named as pathotypes, i.e., pathotype 3 H and 5X, and they were kindly provided by Dr. Strelkov at the University of Alberta. The other type was purified genotypes from *s*ingle-root *p*rotoplast-derived resting spores *i*solates (SPIs) using an unpublished protocol in our group. To generate SPIs, root protoplasts were isolated from small root pieces following cell wall digestion. Resting spores released from every single protoplast were used to infect susceptible control canola plants, and the resulting inoculum from newly formed galls was considered the initial SPIs. These SPIs were then genotyped using Kompetitive Allele Specific PCR (KASP) (LGC Genomics, Teddington, UK), and their virulence races were determined using a set of *B. napus* near-isogenic lines carrying a single clubroot resistance genes (unpublished data). Initial SPIs with distinct differentiation capabilities were selected for use in this study. To improve mapping resolution, the differentiating SPI (i.e., Pb-SPI-117) was used for fine mapping following initial mapping with pathotype 3H. Disease rating was conducted at six weeks post-inoculation (wpi), utilizing a scale of 0 to 3, as described before [40]. Subsequently, the disease severity index (DSI) was determined using an improved methodology [41]. The rating of 0 (no clubbing) was considered resistant (R), and the ratings of 1, 2 and 3 (small, moderate, severe clubs) were susceptible (S). The resistance segregation in the F_2_ and BC_1_ populations was analyzed using a Chi-square Test for the goodness of fit.

### Bulk Segregant RNA-Sequencing (BSR-seq) and sequence analysis

The BC_1_ population was employed for a BSR-seq analysis. At 15 days post-inoculation, an R bulk and an S bulk were formed by combining leaf tissue from 30 resistant and susceptible BC_1_ plants, respectively. One biological replicate consisted of a bulk of resistant or susceptible plants, and three biological replicates were generated per phenotype. RNA was extracted from each bulk, and equal quantities from the three R and three S replicates were pooled separately to construct the pooled sample assembly (PSA) used for BSR-Seq analysis. The RNeasy Plant Mini Kit (Qiagen; Toronto, Canada) was utilized for total RNA extraction from each bulk, with on-column DNAse digestion performed using a Qiagen RNase-Free DNase Set following the manufacturer’s instructions. Assessment of RNA concentration and purity was carried out using a NanoDrop ND-2000c spectrophotometer. The Experion RNA StdSens analysis kit (Bio-Rad; Montreal, Canada) was employed to ensure RNA quality, with a stipulation that the RNA integrity number should exceed 8 for each sample. Subsequent steps in the process included cDNA library preparation following the TruSeq RNA Sample Preparation v2 Guide (Illumina; San Diego, USA). The NanoDrop ND-2000c spectrophotometer was utilized to verify cDNA concentrations and purity. Validation of the cDNA libraries through quality control and qPCR analysis was conducted, and the Experion DNA 1 K Analysis Kit (Bio-Rad) was utilized to confirm their size and purity based on a band at approximately 260 bps. Lastly, RNA-Seq was performed on samples from each inoculated R and S bulk utilizing the Illumina MiSeq platform at the University of Saskatchewan (Saskatoon, Canada).

The high-quality reads obtained from BSR-Seq were mapped to the B genome reference CN115125 v1 (https://cruciferseq.ca/Bnigra*)* using the PSA method [13]. The assembly was conducted with SeqMan NGen 17 software (DNASTAR; Madison, USA). The reference B genome comprises 8 chromosomes, with a combined length totaling approximately 537 million bases (Mb). Subsequently, the short reads generated from the BSR-Seq project were aligned to the individual chromosomes of the reference genome. SNP discovery aimed at marker development was conducted using SeqMan Pro 17 software (DNASTAR), with default settings for SNP discovery parameters, requiring a Q call of ≥ 15 and a depth of ≥ 5. Rough location of the causative gene was determined through analysis of the percentage of polymorphic variants (PPV) [29].

### Single nucleotide polymorphism (SNP) marker genotyping for fine mapping

Selected SNPs identified with BSR-seq were used to design a series of PCR primers and subsequently tested using the KASP (LGC Genomics; Teddington, UK) method following the manufacturer’s instructions. KASP runs were performed on a StepOne Plus Real-Time PCR system (Applied Biosystem; Mississauga, Canada). A total of 1240 plants from the BC_1_ population were used for fine-mapping and inoculated with clubroot isolate Pb-SPI-117. At 6 wpi, leaves from 646 plants with clubroot galls were collected and their extracted total DNA was tested with SNP markers using the KASP method. SNP markers showing the best association pattern with CR phenotype were identified, and in turn, the target region of CR gene on the relevant chromosome of reference B genome could be defined by the location information of those best SNP markers.

### Pan-genomic and phylogenetic analysis for candidate CR genes

All the closely linked SNP markers identified for *Rcr6* [30] and *Rcr12* in this study were searched against the four published reference genomes of *B. nigra*, i.e., CN115125v1, Ni100LRv2.0, Ni100SRv1 [42], and BnSDHv1.1 [43], using the BLAST function on the SequenceServer2.0 of *BnIR*,* Brassica napus multi-omics database (information resource) (hzau.edu.cn)* [44, 45]. The chromosomes harboring the mapped regions (i.e., the targeted regions defined by the closest flanking SNP markers) from each respective reference genome were imported into MegAlign (DNAStar, USA) and aligned using MAUVE algorithm. Further, the mapped regions were used to select target genes, which were then used for functional genomic analysis by OmicsBox (BioBam Bioinformatics, Spain) following the manufacturer’s instructions. After gene annotations, all genes involved in plant immune response were selected as candidate genes.

To check their inner connections, the coding sequences of all the NLR-type candidate genes identified from the mapped region of different reference genomes were compiled and analyzed for phylogenetic distance using MegAlign (DNAStar, USA), and the algorithm used was Maximum likelihood (RAxML). Those confirmed CR genes, such as *CRa*, *Rcr1*, and *Crr1a*, etc., were included for comparison. Since a homologous region on chromosome A08 (14.8–15.4 Mb) was found to share high homology with the *Rcr6* region on chromosome B3 [30], the candidate CR genes from that region were also included in this phylogenetic analysis.

## Results

### Determining the single dominant CR gene status in BRA19278

**Fig. 1:**
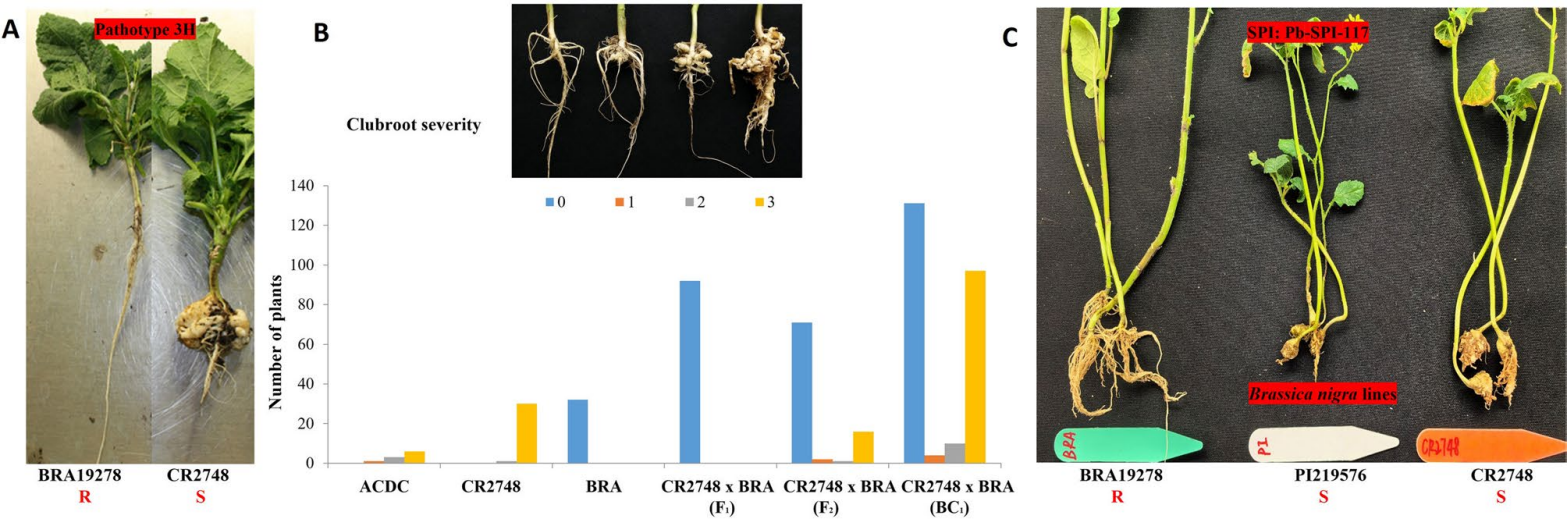
Evaluation of clubroot reaction in *Brassica nigra* lines and descendent populations. **A** Phenotype of resistant (R) line BRA19278 and susceptible (S) line CR2748 to pathotype 3 H. **B** Distribution of phenotypes in *B. nigra* lines and descendent populations to pathotype 3 H. **C** Phenotype of BRA19278, PI219576, and CR2748 to single protoplast isolate (SPI) Pb-SPI-117

When using pathotype 3 H for pathogenicity tests, BRA19278 was found to be highly resistant and no galls were observed in the 36 plants tested (Fig. 1A). In contrast, all of the ACDC and CR2748 plants were susceptible to pathotype 3 H. Complete resistance to pathotype 3 H was found in 92 F_1_ plants derived from the cross between CR2748 and BRA19278 (Fig. 1B), indicating that BRA19278 was likely a homozygous resistant line and the resistance was controlled by dominant gene(s). Further, the segregation ratios for resistance and susceptibility (R: S) in the F_2_ and BC_1_ populations were confirmed by the Chi-Square test to fit well with the expected ratio of 3:1 and 1:1, respectively (Table 1), indicating that CR in BRA19278 is controlled by a single dominant gene, namely *Rcr12*.

**Table 1:** Genetic analysis (Chi-Square test) of clubroot resistance in the segregating populations resulting from the cross between CR2748 (Susceptible, S) and BRA19278 (Resistant, R) and subsequently inoculated with pathotype 3 H of *Plasmodiophora brassicae*

		Phenotype				
Population	Total	Resistant (R)	Susceptible (S)	Expected ratio (R: S)	χ²	*P-value*
BC_1_	242	131	111	1:1	1.65	0.20
F_2_	90	71	19	3:1	0.73	0.39

### Locating the rough region of *Rcr12 *to chromosome B3

**Fig. 2:**
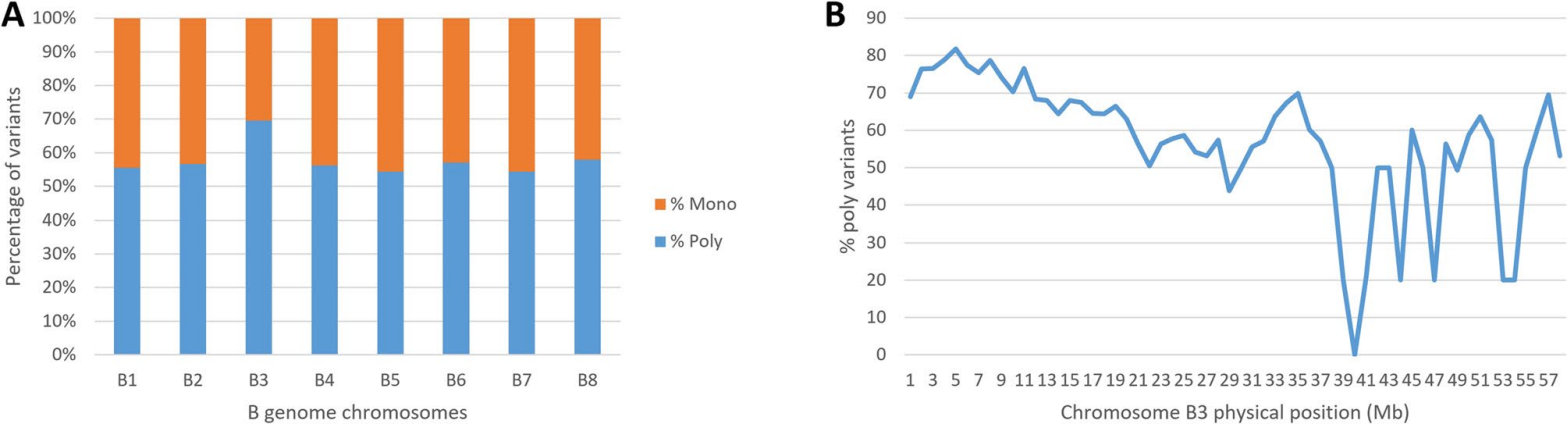
Rough location of *Rcr12* revealed via analysis of polymorphic variants. Bulked segregant RNA sequencing (BSR-seq) data were aligned to reference genome CN115125v1. **A** The percentage of monomorphic and polymorphic variants on each chromosome. **B** The percentage of polymorphic variants on chromosome B3

A mapping-by-sequencing approach via BSR-Seq was performed for BRA19278 because SNP markers linked to *Rcr6* identified from PI219576 were not completely associated with *Rcr12*. To analyze the BSR-Seq data, a pooled sample assembly (PSA), comprising RNA from three biological replicates of R and S bulks, respectively, was used for SNP discovery. The assembly resulted in a total of 27.6 million sequences spanning 4233.7 Mb in length, with 9-fold coverage of the reference B genome, from the pool of three R bulks. Similarly, 37.7 million sequences spanning 4162.9 Mb in length, with 9-fold coverage, were assembled from the pool of three S bulks (Table 2). In total, 2.04 million SNPs were identified upon alignment with the CN115125v1 reference genome. These variants typically encompassed 54–58% polymorphic (poly) variants and 42–46% monomorphic (mono) variants across various chromosomes. Notably, chromosome B3 exhibited a higher PPV (69.6%) and a lower proportion of mono variants (30.4%) compared to other chromosomes (Fig. 2A), suggesting the potential location of *Rcr12* within *B. nigra* chromosome B3.Further analysis of the PPV on chromosome B3 revealed the highest PPV within the physical range of approximately 3.0-7.5 Mb (Fig. 2B), which gave us a rough location to continue our fine-mapping work.

**Table 2:** Short reads from the resistant (R) and susceptible (S) bulks of the BC_1_ population derived from CR2748 x BRA19278 were assembled into *Brassica nigra* reference genome (CN115125v1)

Chromosome number	Chromosome size (bases x 10^6^)	Number of sequences (x 10^6^)		Accumulated length of sequences (bases x 10^6^)	
		R	S	R	S
**B1**	52.4	3.0	5.1	464.9	457.6
**B2**	71.1	3.5	5.8	618.0	606.0
**B3**	58.5	2.9	4.7	516.2	507.7
**B4**	57.8	2.5	4.2	514.5	506.2
**B5**	57.4	2.6	4.4	505.8	497.3
**B6**	60.2	3.1	5.2	531.3	522.4
**B7**	52.3	2.1	3.5	468.8	461.6
**B8**	68.8	2.9	4.8	614.2	604.1
**Total**	478.5	27.6	37.7	4233.7	4162.9

### Selection of differentiating strain for fine mapping

In our screening for CR materials in different *B. nigra* lines, BRA19278 and PI219576 both showed good resistance to pathotypes in Canada, such as pathotype 3 H, 5X, etc. Based on the gene-for-gene theory [46], we speculated that the observed broad spectrum of resistance in multiple *B. nigra* materials should be attributed to more than one CR genes. Thus, a differentiating strain that could separate BRA19278 from PI219576 is critical for our cloning/characterization work on CR genes from *B. nigra*. After obtaining more than 100 SPIs in our lab (unpublished data), we conducted a thorough screening of the two *B. nigra* lines. Luckily, Pb-SPI-117 was confirmed to be able to induce large galls in PI219576, the *Rcr6* donor, while failing to do so in BRA19278 (Fig. 1C). The distinct and significant differences between BRA19278 and PI219576 observed in repeated inoculation tests indicated that a novel CR gene may be present in BRA19278 (Table 3). To obtain higher-resolution phenotypic data for fine mapping of *Rcr12*, the differentiating SPI Pb-SPI-117 was used to inoculate the same segregating populations (Fig. 1C). Complete resistance in the F_1_ plants and segregation ratios in the F_2_ and BC_1_ generations again fit the expected 3:1 and 1:1 models, further supporting the single dominant gene hypothesis.

**Table 3:** Results summary of pathogenicity tests with different inoculums of *Plasmodiophora brassicae*

		Disease Severity Index (DSI) with single protoplast isolates						DSI with field collected pathotypes	
Plant	CR gene candidates	Pb-SPI-117	Pb-SPI-151	Pb-SPI-135	Pb-SPI-170	Pb-SPI-65	Pb-SPI-55	3H	5X
**BRA19278**	***Rcr12***	0 ± 0	0 ± 0	0 ± 0	0 ± 0	0 ± 0	0 ± 0	0 ± 0	0 ± 0
**PI219576**	***Rcr6***	82.2 ± 16.7	0 ± 0	0 ± 0	0 ± 0	0 ± 0	0 ± 0	0 ± 0	0 ± 0
**CR2748**	**N/A**	100 ± 0	100 ± 0	100 ± 0	100 ± 0	100 ± 0	100 ± 0	97.2 ± 2.4	94.4 ± 6.4
**ACDC**	**N/A**	100 ± 0	100 ± 0	100 ± 0	100 ± 0	100 ± 0	100 ± 0	82.7 ± 6.8	94.4 ± 6.4
**Y549-(0)−2-1**	***Rcr1***	100 ± 0	100 ± 0	100 ± 0	100 ± 0	100 ± 0	0 ± 0	0 ± 0	100 ± 0
**HH393-2**	***Rcr1***	100 ± 0	100 ± 0	100 ± 0	100 ± 0	100 ± 0	0 ± 0	0 ± 0	100 ± 0
**DH16516**	**N/A**	100 ± 0	100 ± 0	100 ± 0	100 ± 0	100 ± 0	100 ± 0	100 ± 0	100 ± 0
**DH12075**	**N/A**	100 ± 0	100 ± 0	100 ± 0	100 ± 0	100 ± 0	100 ± 0	100 ± 0	100 ± 0

### Fine-mapping *Rcr12 *to a 0.33 Mb region on chromosome B3

**Fig. 3:**
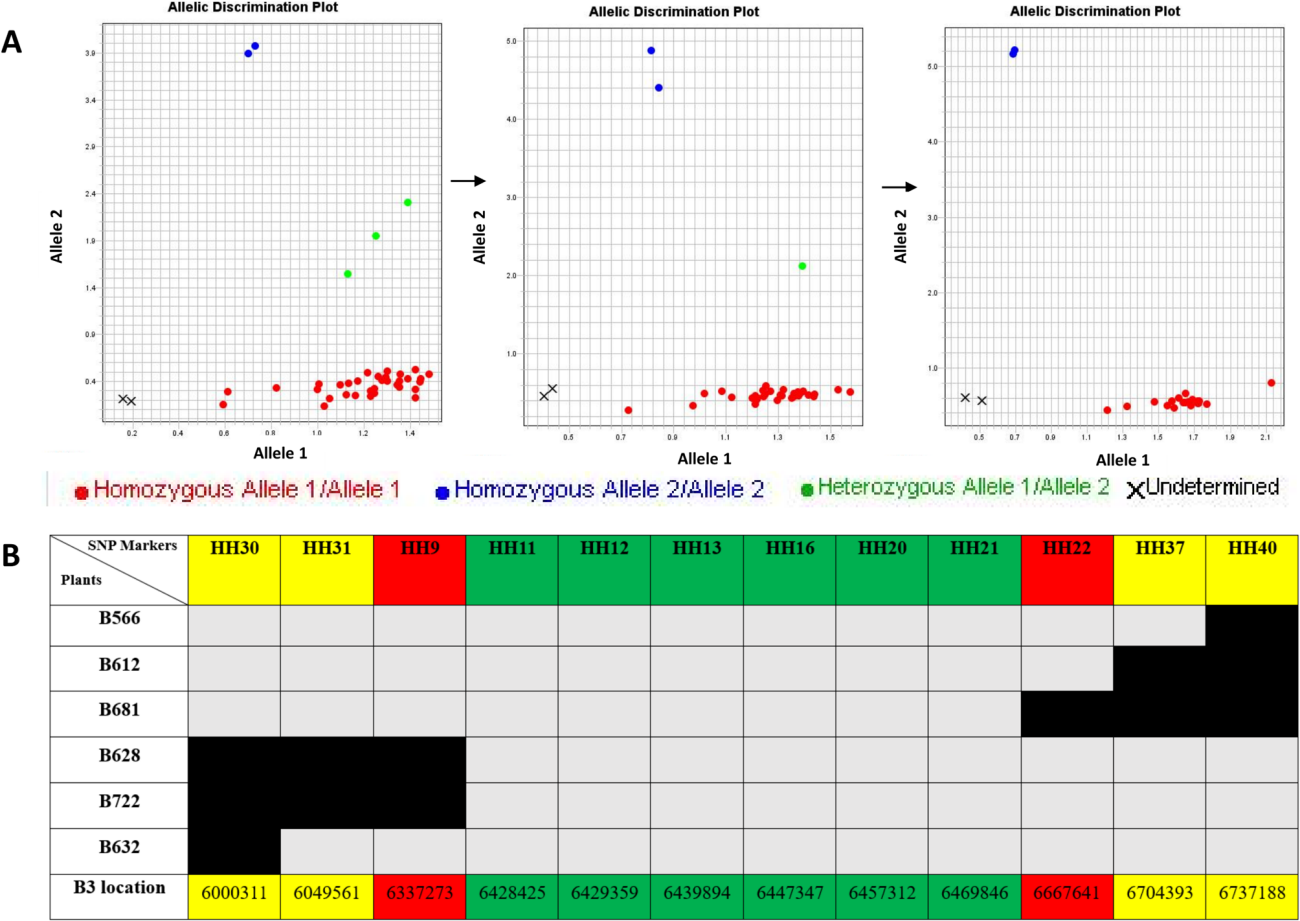
Fine-mapping of *Rcr12* to a 0.33 Mb region on chromosome B3. A total of 1240 plants from the BC_1_ population (CR2748 x BRA19278) were inoculated with the SPI Pb-SPI-117, and DNA extracted from 648 susceptible plants were used for fine-mapping. **A** Genotyping with Kompetitive Allele Specific PCR (KASP) using newly designed SNP markers. **B** A 0.33 Mb region on chromosome B3 was defined as the target region of *Rcr12* by best SNP markers

Based on the polymorphic variants identified from the rough location on chromosome B3, a series of SNP markers covering the entire region (i.e., 3.0-7.5 Mb) were designed as KASP primers and tested in KASP genotyping runs to screen for recombinant events in the 646 susceptible plants from the BC_1_ population (Fig. S1). SNP markers with the best performance (i.e., good separation and association pattern with phenotypes) were used to define a further-refined region, which was then used to start a new round of screening process. Starting from about 50 recombinant events in the tested population, new SNP markers designed from the further refined region showed a clear pattern toward fewer and fewer recombinant events identified from each side until no recombinant could be identified (Fig. 3A). After three rounds of screening, 6 SNP markers were found to be completely associated with the phenotype observed from the tested population of 1240 plants; thus, a 0.33 Mb region was defined by the two adjacent SNP markers at the left and right border, i.e., HH9 (B3 location: 6337273) and HH22 (B3 location: 6667641) (Table S1, Fig. 3B).

### Pan-genomic and phylogenetic analyses confirmed *Rcr12 *as a distinct CR gene

**Fig. 4:**
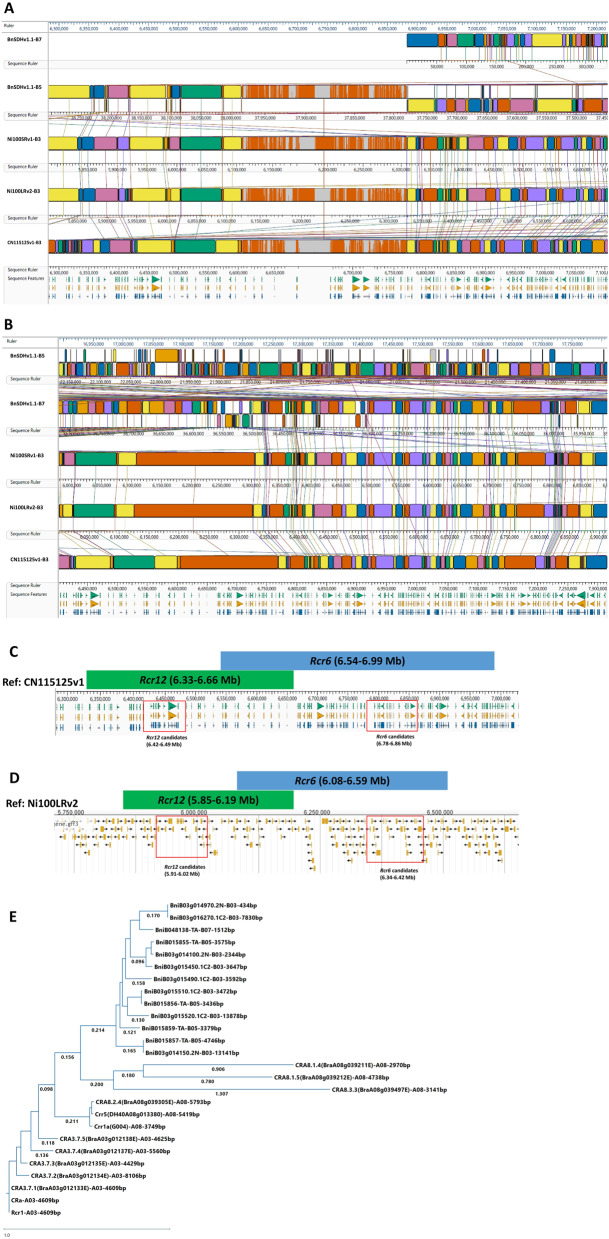
Pan-genomic and phylogenetic analysis of *Rcr12* and *Rcr6*. Four available reference genomes of *B. nigra*, BnSDHv1.1, Ni100SRv1, Ni100SRv2, and CN115125v1, were all used in this study. **A** Targeted regions of *Rcr12* in different reference genomes. **B** Targeted regions of *Rcr6* in different reference genomes. **C** Target regions and candidate CR genes for *Rcr12* and *Rcr6* in reference genome CN115125v1. **D** Target regions and candidate CR genes for *Rcr12* and *Rcr6* in reference genome Ni100LRv2. **E** Phylogenetic analysis of 13 candidate NLR type genes of *Rcr12/Rcr6* and other CR genes from chromosome A08 and A03. Gene sequences from B genome were obtained from each relevant reference genome of *B. nigra*, i.e., BnSDHv1.1 (gene names ended with *-TA*), Ni100SRv2 (gene names ended with *0.2 N*), and CN115125v1 (gene names ended with *.1C2*). Gene sequences of *CRAx.x.x* were obtained from ECD04 (*B. rapa*) reference genome. Other CR gene sequences from chromosome A08 or A03 (i.e., *Rcr1*, *CRa*, *Crr1a*, *Crr5*) were obtained from BLAST search in NCBI

BLAST search using all the closely linked SNP markers with *Rcr12* or *Rcr6* identified different target regions from each of the four reference genomes of *B. nigra*. In the BnSDHv1.1 reference genome [43], *Rcr12-*related SNP markers were found to cluster on the 37.9 ~ 38.2 Mb region of Chromosome B5 (Fig. 4A), while *Rcr6-*related SNP markers clustered on the 36.3 ~ 36.6 Mb region of Chromosome B7 (Fig. 4B; Table 4). This is another clear evidence that *Rcr12* and *Rcr6* are two different CR genes from the B genome. Further, for the other three reference genomes of CN115125v1/Ni100LRv2.0/Ni100SRv1 [42], the mapped regions of *Rcr12* and *Rcr6* were all on chromosome B3, and they overlapped slightly with each other. However, the two mapped regions of *Rcr12* and *Rcr6* on chromosome B3 showed high synteny with chromosome B5 and B7 in BnSDHv1.1, respectively, but the overlapped region only showed high homology with chromosome B5, no homology with chromosome B7 in BnSDHv1.1 at all (Fig. 4A and B). This indicated that the overlapped region was supposed to be only part of the mapped region of *Rcr12*, not to be shared with the mapped region of *Rcr6*. Besides, the distinct identities of the two mapped CR genes were demonstrated by the different candidate genes identified from each mapped region (Fig. 4C; Table 4). For example, on the reference genome of CN115125v1, *Rcr12* was mapped to the 6.35 ~ 6.66 Mb region of chromosome B3, which shared a 0.12 Mb of overlapped region (i.e., 6.54 ~ 6.66 Mb) with the mapped region of *Rcr6* (6.54 ~ 6.99 Mb on Chromosome B3) (Fig. 4C). However, after gene annotation, results showed that all the disease-resistance related candidate genes for *Rcr12* were clustered around 6.42 ~ 6.49 Mb on chromosome B3 of CN115125v1, while candidates for *Rcr6* were clustered around 6.78 ~ 6.86 Mb, i.e., a physical distance of 0.29 Mb between the two mapped CR genes. Similar phenomena could also be observed in the two versions of Ni100 reference genomes (Fig. 4D; Table 4). Another significant contribution of the pan-genomic analysis was to identify more CR candidates for the cloning/characterization work. In total, 10 and 3 NLR-type genes were identified for *Rcr12* and *Rcr6*, respectively, from the four reference genomes (Only results from three reference genomes are listed in Table 4, since two reference genomes, i.e., Ni100LRv2.0/Ni100SRv1, were both from the same *B. nigra* material and gave the same results in our pan-genomic analysis). The interconnections among these B3 NLRs as well as other CR genes identified from chromosomes A08 and A03 were further checked using phylogenetic analysis based on the comparison of their coding sequences.

**Table 4:** Candidate genes identified from targeted regions of relevant reference genomes

Reference genome	CR gene	Target region (Mb)	Candidate CR genes	Start location	End location	Size (bp)	Type	Description
**CN115125v1**	*Rcr12*	B3: 6.33 ~ 6.66	BniB03g015450.1C2	6,426,485	6,430,131	3646	TNL	Probable disease resistance protein RPP1
			BniB03g015490.1C2	6,439,814	6,443,405	3591	TNL	Probable disease resistance protein RPP1
			BniB03g015510.1C2	6,447,293	6,450,764	3471	TNL	Probable disease resistance protein RPP1 isoform X1
			BniB03g015520.1C2	6,456,006	6,469,883	13,877	TNL	Probable disease resistance protein RPP1
			BniB03g015570.1C2	6,497,781	6,498,038	257	-	PAMP-induced secreted peptide 2-like involved in innate immune response
	*Rcr6*	B3: 6.54 ~ 6.99	BniB03g016100.1C2	6,784,057	6,784,437	380	-	Defensin-like protein 75
			BniB03g016110.1C2	6,789,037	6,789,441	404	-	Defensin-like protein 75
			BniB03g016270.1C2	6,852,715	6,860,544	7829	TNL	Probable disease resistance protein RPP1
**Ni100LRv2**	*Rcr12*	B3: 5.85 ~ 6.19	BniB03g014100.2 N.1	5,916,497	5,918,840	2343	TNL	Probable disease resistance protein RPP1
			BniB03g014150.2 N.1	5,958,787	5,971,927	13,140	TNL	Probable disease resistance protein RPP1
			BniB03g014250.2 N.1	6,027,415	6,027,621	206	-	PAMP-induced secreted peptide 2-like involved in innate immune response
	*Rcr6*	B3: 6.08 ~ 6.59	BniB03g014800.2 N.1	6,341,855	6,342,235	380	-	defensin-like protein 75
			BniB03g014820.2 N.1	6,351,736	6,352,140	404	-	defensin-like protein 75
			BniB03g014970.2 N.1	6,424,432	6,424,865	433	NLR	disease resistance protein TAO1-like
**BnSDHv1.1**	*Rcr12*	B5: 37.9 ~ 38.2	BniB015855-TA	38,169,257	38,172,831	3574	TNL	probable disease resistance protein RPP1
			BniB015856-TA	38,154,453	38,157,888	3435	TNL	probable disease resistance protein RPP1 isoform X1
			BniB015857-TA	38,147,255	38,152,000	4745	TNL	probable disease resistance protein RPP1
			BniB015859-TA	38,132,278	38,135,656	3378	TNL	Disease resistance protein (TIR-NBS class)
	*Rcr6*	B7: 36.2 ~ 36.6	BniB015822-TA	36,651,147	36,654,300	3153	-	thaumatin-like protein 1 involved in defense response
			BniB048138-TA	36,400,810	36,402,321	1511	TNL	probable disease resistance protein RPP1 isoform X1

In an overview, the phylogenetic analysis has demonstrated a clear separation of the three clusters of NLR genes from three different chromosomes (i.e., B3, A03, and A08). The grouping pattern of the NLR genes of three chromosomes agreed with their different origins and showed their evolutionary distances. The target region mapped on chromosome B3 did share higher homology with the relevant region on chromosome A08 compared to the region on chromosome A03 that contains the NLR cluster of *CRa*/*Rcr1*. Furthermore, the phylogenetic analysis confirmed the similar pattern of NLR clusters on the four different reference genomes (Table 4). As shown in Fig. 4E, each of those NLRs in order is quite similar to the corresponding one in the relevant reference genome. For example, *BniB03g015450.1C2* and *BniB03g015510.1C2* are quite close to *BniB015855-TA* and *BniB015856-TA* in their coding sequences, respectively. Similar NLR clusters with right-sized genes (around 4 kb) are presented in different *B. nigra* materials, it is highly likely these NLR genes are all CR genes and responsible for the strong yet still race-specific type of resistance observed. Regarding candidate CR genes for *Rcr6*, from a conservative perspective, the ideal candidate gene would be *BniB03g016270.1C2* since it is the only NLR-type with the right domain structure available, while the other two candidate genes (i.e., *BniB03g014970.2 N* and *BniB048138-TA*) are not ideal due to size limitations and further analysis on their domain structures. Nevertheless, other types of disease-related genes predicted in the fine-mapped regions, for example, defensin-like proteins, could also be *Rcr12*/*Rcr6* candidates and worth further investigation.

## Discussion

In the Brassicaceae family, screening for CR materials has revealed that CR resources from *B. nigra* lines (B genome) are relatively abundant. This is unsurprising, as many *B. nigra* accessions exhibit wild plant characteristics, which are often associated with a higher density of *R* genes compared to domesticated crops [47]. However, despite the richness of CR resources in the B genome, the number of reported CR genes from B genome remains limited, highlighting a lack of systematic exploration and utilization. Given the considerable challenges, if not impossible, to introgress CR traits from near-wild *B. nigra* plants into canola [48], CR genes identified in the B genome hold greater breeding value compared to those from the A genome. This underscores the importance of further investigation into the B genome as a potential reservoir of valuable CR genes.

In this study, we successfully identified *Rcr12* in the B genome, a single dominant CR gene located in close proximity to, but distinct from, the previously reported *Rcr6*. First of all, the fine mapping work of *Rcr12* started from a distinct phenotype of full resistance of BRA19278 against a differentiating SPI Pb-SPI-117, which is a clear contrast to the susceptible phenotype observed in PI219576 (Fig. 1A). The strong CR phenotype observed in BRA19278 against Pb-SPI-117 was later demonstrated to be controlled by a single dominant gene. Logically, the single dominant CR gene responsible for this superior resistance in BRA19278 should not be present in PI219576 which carries *Rcr6*. Conversely, whether *Rcr6* is present in BRA19278 is not definitive since BRA19278 is also resistant to pathotype 3 (the inoculum that helped identify *Rcr6* from PI219576), while the SNP markers closely linked to *Rcr6* were not performing well in BRA19278-derived populations. Secondly, the new SNP markers developed in this study for *Rcr12* worked well in BRA19278 populations, but not so well in PI219576, indicating some major differences/variations in the targeted region of the two *B. nigra* lines. Further, in pan-genomic analysis, when using different reference genomes, a clear separation of the clustering candidate CR genes in either of the targeted regions of *Rcr12* or *Rcr6* was observed (Fig. 4C and D; Table 4). We note that structural variation across *Brassica* reference genomes can lead to differences in chromosome assignments and interval boundaries. For example, the *Rcr12* locus mapped to chromosome B5 in BnSDHv1.1, rather than B3 as in the other two reference genomes. Despite this structural discrepancy, the gene content within the corresponding interval supports the functional distinction between *Rcr12* and *Rcr6*, thereby reinforcing our conclusion. Such pangenomic differences highlight the importance of using multiple reference genomes to capture structural and genic diversity. Based on the definition of *R*-gene clustering, i.e., within 200 kb of distance and fewer than eight non-NLR genes [33, 49], it’s obvious that *Rrc12* and *Rcr6* belonged to two different *R*-gene loci. More specifically, *Rcr12* represents an NLR cluster, while *Rcr6* is an NLR singleton. It is generally considered that NLR clusters are more complex NLR systems that may have evolved from NLR singletons [34]. The combination of an NLR cluster (*Rcr12*) and an NLR singleton (*Rcr6*) in close proximity on chromosome B3 (Fig. 4C and D; Table 4) represents the first report of this type of NLR organization in *Brassica* crops. Given their sequence similarity (Fig. 4E) and close physical location, the functional and potential evolutionary connections between these two CR gene loci merit further investigation. Overall, *Rcr12* is the second CR gene identified from B genome.

Regarding CR gene clustering on chromosome B3 observed in this study, it is a new example in addition to other CR gene clusters found in A genome like *CRa/CRb/Rcr1/Rcr2/BraA.CR.a/CRA3.5/CRA3.6/CRA3.7* in chromosome A03 and *Crr1/CRs/PbBa8.1/Rcr9/Rcr3/BraA.CR.b/CRA8.1/CRA8.2/CRA8.3* in chromosome A08 [32]. It is generally accepted that the clustering of NLR type of *R* genes is evolutionarily more beneficial for the fitness of plants against biotic diseases [33, 34, 49–51]. For example, growing evidence suggests that NLR clusters’ gene products can cooperate, such as through homo-/hetero-dimerization or oligomerization, to trigger immune responses. This could result in massive expansions on the pathogen recognition possibilities using a limited number of genes within the cluster. Also, close genomic proximity within NLR clusters may prevent recombination and separation of co-functioning NLRs, further enhancing coregulatory benefits in evolution. However, the role of each *R* gene, NLR type or not, in this clustering genomic structure is still largely unclear, and may turn out to be in a case-by-case situation. The pattern and individual roles of these typical clustering *R* genes merit thorough investigation, as such insights could significantly enhance the efficiency of efforts to characterize the contributions of each gene within this coordinated group response.

Nonetheless, before deciphering the secret code behind the clustering phenomenon, at this stage, some other routes could also help identify more CR genes, for example, the availability of high quality reference genomes of elite CR materials, and a unified classification system on the pathotype/isolate of pathogen *P. brassicae*. Currently, there are some confusions/ambiguities in the field of clubroot research especially the identification of CR genes. This is largely caused by the use of different CR plant materials with related but distinct genomic/genetic backgrounds (for example, *B. rapa* versus *B. nigra* plants), and a non-unified classification system of pathogen pathotypes. Different CR materials are found from all over the world that grows *Brassica* crops, and their genomic backgrounds are hard to compare without the support of high quality reference genomes. Regarding the protist pathogen, due to its fastidious nature, pure culture is still not available and thus most clubroot researchers are forced to work with field-collected pathotypes with a mixed nature. This is clearly a cause of uncertainty in obtaining any definitive results with great potential of chaotic consequences. With the current unpurified pathotypes prevailing in most clubroot labs, it adds another layer of uncertainty to compare mapping results from different research groups, thus the ambiguous identities of CR genes reported in the last three decades arise. Nowadays, with the help of fast evolving next-generation-sequencing (NGS) technologies, high quality plant genomes are produced on a daily basis, thus reference genomes are becoming less and less a bottleneck for researchers. While on the other hand, this has further enhanced the urgent needs for a better classification system of clubroot pathogen.

Mutations and diversifications are the natural abilities for living creatures to survive and evolve under various biotic and abiotic selection pressures. This rich diversity is critical and necessary for the endurance of any species. However, it also hurdles the investigations into one-on-one type of interactions such as gene-for-gene in classical plant disease studies. Considering the relatively longer life cycles of plants, it is easier to obtain and keep purer breeding lines using technologies like double haploidy in canola. On the other hand, pathogenic microorganisms, especially those fastidious/uncultured ones, are notorious for their mixed identities in naturally obtained isolates (NOIs). The interactions observed between various in-house plant materials and NOIs are usually hard to interpret or even repeat due to the dynamic identities in NOIs. In turn, this has greatly impeded the discovery of individual CR genes especially when clustering of CR genes is a common phenomenon in Brassicae plants after the ancient genome triplication event. The ability to use pure or near pure isolates for clubroot research is a critical and initial step in obtaining any meaningful results. Our successful identification of a novel CR gene in such close proximity to *Rcr6* can be largely attributed to the pioneering use of SPIs in this study, a unique and powerful resource. Looking ahead, we could anticipate that the discovery of novel CR genes and other relevant investigations in this field will be greatly accelerated after the establishment and release of the new SPI system of *P. brassicae* in the near future.

## Supplementary Information

Supplementary Material 1: Table S1 List of SNP marker sequences identified in the target region of Rcr12 on the chromosome B3 of Brassica nigra line BRA19278.

Supplementary Material 2: Fig. S1 Schematic distribution of SNP markers used for fine mapping of Rcr12 on chromosome B3 (CN115125v1). Markers are plotted according to their physical positions along the B3 chromosome. Polymorphic SNP markers (green dots) are labeled with their marker names and positions; monomorphic SNP markers (gray dots) are shown as unlabeled dots for clarity. Two border-defining polymorphic markers, HH9 and HH22, flanking the finely mapped core region, are highlighted in red. This figure provides an overview of marker distribution and polymorphism patterns used in narrowing down the Rcr12 candidate interval.

## Data Availability

All data supporting the findings of this study are provided within the paper, and its supplementary materials are published online. The RNA-sequencing datasets generated during the current study are available in the GenBank repository under BioProject accession PRJNA1243463 at PRJNA1243463.

## Abbreviations

BSA: Bulk segregant analysis
BSR-Seq: Bulked segregant RNA sequencing
CNL: Coiled-coil (CC)-NB-LRR
CR: Clubroot resistance
DSI: Disease severity index
KASP: Kompetitive Allele Specific PCR
NGS: Next-generation sequencing
NLR: Nucleotide-binding leucine-rich repeat (NB-LRR)
NOI: Naturally obtained isolate
PAMP: Pathogen-associated molecular pattern
PPV: Percentage of polymorphic variants
PSA: Pooled sample assembly
PTI: PAMP-triggered immunity
QTL: Quantitative trait locus
R: Resistant
Rcr12: Resistance to clubroot 12
Rcr6: Resistance to clubroot 6
RLP: Receptor-like protein
RLK: Receptor-like kinase
S: Susceptible
SNP: Single-nucleotide polymorphism
SPI: Single-protoplast isolate
TIR: Toll/interleukin-1 receptor
TNL: Toll/interleukin-1 receptor-NB-LRR
