# Supplementary material for: Identification of Rcr12, a single dominant clubroot resistance gene near Rcr6 on chromosome B3 of Brassica nigra
Source: BMC Plant Biol. 2025 Jul 18;25:925. doi: 10.1186/s12870-025-06947-3 (PMC12273221; doi:10.1186/s12870-025-06947-3)
Supplement: Supplementary file 1 — Supplementary Material 1: Table S1 List of SNP marker sequences identified in the target region of Rcr12 on the chromosome B3 of Brassica nigra line BRA19278. [file 12870_2025_6947_MOESM1_ESM.docx]

**Table S1** List of SNP marker sequence identified in the flanking region of *Rcr12* on the chromosome B3 of *Brassica nigra* line BRA19278 (reference genome CN115125v1)

| **SNP marker** | **B3 location** | **KASP primer sequence (FAM/HEX/COM)** |
| --- | --- | --- |
| **HH11** | 6428425 | **GAAGGTGACCAAGTTCATGCT**ACAACCACCAAATGAGATTTCTTTTCATCC |
|  |  | **GAAGGTCGGAGTCAACGGATT**CACAACCACCAAATGAGATTTCTTTTCATCT |
|  |  | CAACAGTAAATTGGAACCATCAACTTAACTC |
| **HH12** | 6429359 | **GAAGGTGACCAAGTTCATGCT**TCCTCCATCACCAGCTTTTCAGC |
|  |  | **GAAGGTCGGAGTCAACGGATT**CTCCTCCATCACCAGCTTTTCAGT |
|  |  | TTGCTGACTGCGAAGGAACAAGAC |
| **HH13** | 6439894 | **GAAGGTGACCAAGTTCATGCT**CTGCTGCCATCAGCTTCTACGCT |
|  |  | **GAAGGTCGGAGTCAACGGATT**TGCTGCCATCAGCTTCTACGCC |
|  |  | GTTTTGTTTTCTTGATGAGATTTGGAAGATTTTCTG |
| **HH16** | 6447347 | **GAAGGTGACCAAGTTCATGCT**ATCCTCCTATTTTCATCATACTAATGGATTCG |
|  |  | **GAAGGTCGGAGTCAACGGATT**GATCCTCCTATTTTCATCATACTAATGGATTCT |
|  |  | CTGATGAAAAAAATTGTAGCCAAGAGAGC |
| **HH20** | 6457312 | **GAAGGTGACCAAGTTCATGCT**CTGGGAGTTCCTTGACGGTGG |
|  |  | **GAAGGTCGGAGTCAACGGATT**CTTCTGGGAGTTCCTTGACGGTGA |
|  |  | CCATTTCGTCCTCACCACCACATAC |
| **HH21** | 6469846 | **GAAGGTGACCAAGTTCATGCT**CGAACGTAACGATAAAAGAATGCGGGATAT |
|  |  | **GAAGGTCGGAGTCAACGGATT**GAACGTAACGATAAAAGAATGCGGGATAC |
|  |  | CTCAATGCCTAAAGGCTCATAATCTTCATAGC |
