# Supplementary figures and images for: Identification of Rcr12, a single dominant clubroot resistance gene near Rcr6 on chromosome B3 of Brassica nigra
Source: BMC Plant Biol. 2025 Jul 18;25:925. doi: 10.1186/s12870-025-06947-3 (PMC12273221; doi:10.1186/s12870-025-06947-3)

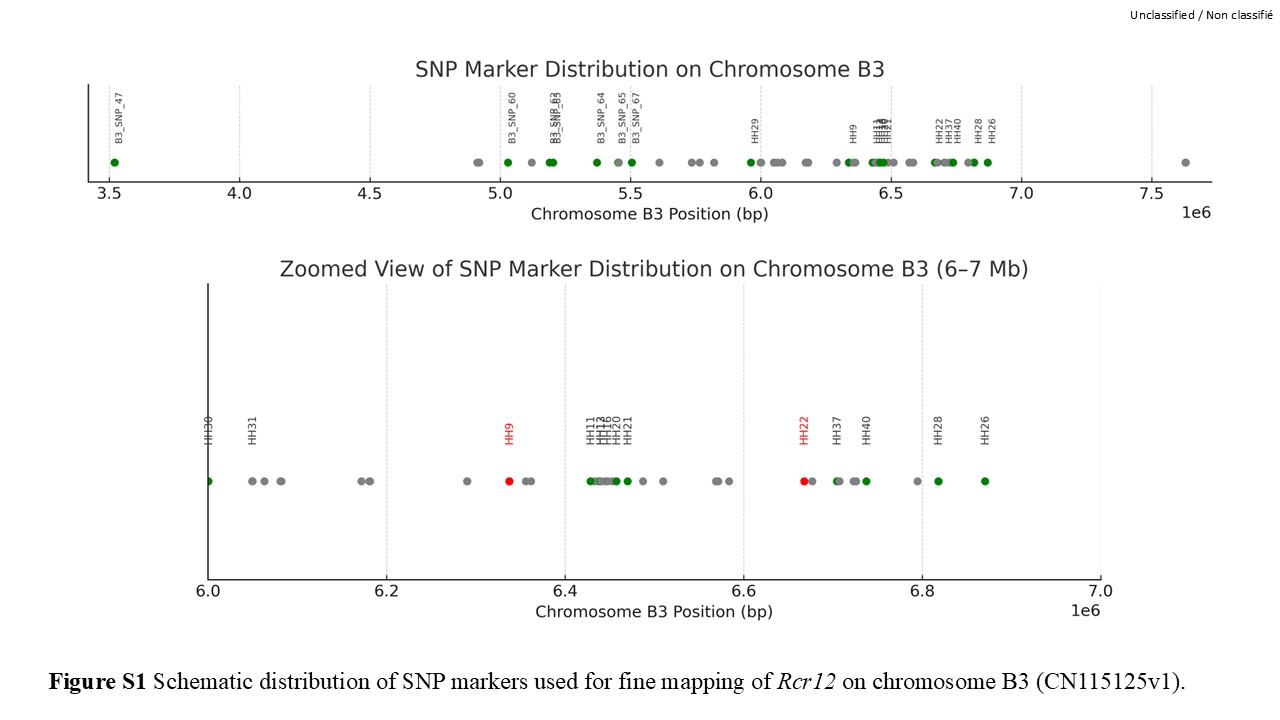

Supplement: Supplementary file 2 — Supplementary Material 2: Fig. S1 Schematic distribution of SNP markers used for fine mapping of Rcr12 on chromosome B3 (CN115125v1). Markers are plotted according to their physical positions along the B3 chromosome. Polymorphic SNP markers (green dots) are labeled with their marker names and positions; monomorphic SNP markers (gray dots) are shown as unlabeled dots for clarity. Two border-defining polymorphic markers, HH9 and HH22, flanking the finely mapped core region, are highlighted in red. This figure provides an overview of marker distribution and polymorphism patterns used in narrowing down the Rcr12 candidate interval. [file 12870_2025_6947_MOESM2_ESM.jpg]
